# Statistical Approaches to Use a Model Organism for Regulatory Sequences Annotation of Newly Sequenced Species

**DOI:** 10.1371/journal.pone.0042489

**Published:** 2012-09-11

**Authors:** Pietro Liò, Claudia Angelini, Italia De Feis, Viet-Anh Nguyen

**Affiliations:** 1 Computer Laboratory, University of Cambridge, Cambridge, United Kingdom; 2 Istituto per le Applicazioni del Calcolo “Mauro Picone” (CNR), Napoli, Italy; National Taiwan University, Taiwan

## Abstract

A major goal of bioinformatics is the characterization of transcription factors and the transcriptional programs they regulate. Given the speed of genome sequencing, we would like to quickly annotate regulatory sequences in newly-sequenced genomes. In such cases, it would be helpful to predict sequence motifs by using experimental data from closely related model organism. Here we present a general algorithm that allow to identify transcription factor binding sites in one newly sequenced species by performing Bayesian regression on the annotated species. First we set the rationale of our method by applying it within the same species, then we extend it to use data available in closely related species. Finally, we generalise the method to handle the case when a certain number of experiments, from several species close to the species on which to make inference, are available. In order to show the performance of the method, we analyse three functionally related networks in the *Ascomycota*. Two gene network case studies are related to the G2/M phase of the *Ascomycota* cell cycle; the third is related to morphogenesis. We also compared the method with MatrixReduce and discuss other types of validation and tests. The first network is well known and provides a biological validation test of the method. The two cell cycle case studies, where the gene network size is conserved, demonstrate an effective utility in annotating new species sequences using all the available replicas from model species. The third case, where the gene network size varies among species, shows that the combination of information is less powerful but is still informative. Our methodology is quite general and could be extended to integrate other high-throughput data from model organisms.

## Introduction

One of the most important and time consuming step in annotating a new genome is the identification of the transcription factor binding sites [1], [2]. An important reason for such difficulty is their fast evolution with respect to coding regions, which limits the use of model organisms annotation [3]. Recently, due to the direct sequencing of all DNA fragments from ChIP assays, ChIP-Seq has become the best technology for genome-wide mapping of protein-DNA interactions [4].

An important class of binding site identification methods is based on the assumption that co-expressed groups of genes often share regulatory elements, which mediate the co-expression; interesting counter examples are described in [5]. A two-step approach is most commonly used. In the first step, the co-expressed groups of genes need to be determined, typically from gene-expression data. A clustering procedure is performed to partition the genes into groups believed to be co-regulated, based on expression profile similarity. In the second step, a motif discovery tool is applied to search for abundant sequence patterns in the promoters (or 3′-UTRs) of each group that may represent the binding sites of transcription factors that regulate the corresponding genes. In [6] the authors applied linear regression with stepwise selection on a list of candidate motifs obtained using MDScan (see [7]) which is an algorithm that makes use of word-enumeration and position-specific probability matrix updating techniques. The candidate motifs were scored in terms of number of sites and degree of matching with each gene. Inspired by Liu's work, our group has explored the performances of algorithms based on Bayesian variable selection techniques showing that they can be more effective than stepwise regression [8],[9],[10]. In particular, in [10] and [8] we described a Bayesian variable selection model to take into account the different and multiple information sources available, to pool together results of several experiments and to allow the users to select the motifs that best explain and predict the changes in expression level in a group of co-regulated genes. When experiments are costly, particularly in high throughput biology, replicates come often in a minimum number to assure statistical reliability for disseminating and publishing results. In some cases, recently diverged species might retain similarities in gene expression. These considerations suggest that, in absence of experimental replicates, or even in addition to these, statistical support to experimental evidences may also be obtained by analysing model organisms that are phylogenetic close variants of the species under examination. The effective exploitation of annotated species richness is hampered by the lack of a robust theoretical statistical framework to combine and contrast the knowledge from replicas and from the model organisms nearby species. Here we describe a new systematic genome-wide statistical approach for identifying putative transcription factor binding sites from over-represented DNA sequence elements, or motifs, of newly sequenced species, by regressing gene expression data of nearby model species. The phylogenetic relationship between the species, using coding regions, is carried out for the sole purpose of identifying those model species that are enough close to potentially share similarity in gene expression and motifs. Then we use Bayesian variable selection to combine the information of the DNA sequences of the species under analysis with the genome expression information of other sufficiently close species, from which several experimental results are available. Pooling information across studies can help to accurately identify the true target genes, as pointed out in [11], allowing both to share the final cost of the analysis and to use already available data which are contained in classical repositories. The paper is organized as follows. First we set the rationale of our method by applying it within the same species, then we extend it to use data available in closely related species. With respect to previous publications, here we present a general algorithm to identify transcription factor binding sites in one species and perform Bayesian regression on the annotated species. Our generalisation could handle the case when a certain number of experiments from several species closed to the species on which to make inference are available. We also introduce an internal testing analysis and we investigate three different networks; then we compare results with those obtained using MatrixReduce, one of the best performing and used algorithm in the field [12]. Finally we discuss the findings on the three networks and we describe the statistical methodology in the Section Methodology.

## Materials and Methods

### Algorithm

The algorithm consists of three major stages: sequence processing, candidate motif selection, and motif detection. In the following subsections we describe the specific steps we carried out.

### Sequence Preprocessing Steps

1) Select a group of co-regulated genes in a well-annotated species and collect related microarray expression experiments. In our study we considered three case studies with different model organisms: the septation transcriptional network in *S. Pombe*; the cytokinesis transcriptional network and RAM signalling network, both in *C.albicans*.

2) Determine the nearby species using phylogenetic properties of the gene set selected in the previous step. Phylogenetic analysis can be conducted with several methodologies. In the first case study, we assessed the distance among fungi species based on all Ace2p related protein trees using the JTT amino acid substitution model [13]. The choice was due to the fact that these proteins are globular cytoplasmic proteins. Likelihood maximization and maximum likelihood parameter estimation were performed by numerical optimization routines using a single replacement matrix for all sites. Based on phylogenetic similarity we selected *S. Japonicus* and *S. Octosporus* as nearby species of *S. Pombe*. In the second case study, we generated all RAM related trees and picked three candida genomes *C. Tropicalis*, *C. Dublinensis* and *C. Parapsilosis* as nearby species of *C. Albicans*. Note that the latter two species seem to be more distant from the first two species.

3) Choose a set of biologically independent genes for each model species (*S. Pombe* 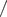 *C. Albicans*) from the pool of remaining genes (those not selected in step (1)). This step is motivated from the fact that extensive comparative genomic analysis has revealed that all the eukaryotic genomes contain families of duplicated genes which have recently diverged. In many cases these families have retained large part of the upstream regulatory sequences. In particular the residues of whole genome duplications have been identified in different yeast strains [14] as well as in other species. The redundancy of yeast genome suggests us to select a meaningful non redundant ensemble of genes that contains all the relevant statistical characteristics of the genome and therefore will play the role of control genes in step (6).

To this purpose, we performed a phylogenetic analysis of the gene pool using standard maximum likelihood techniques. The analysis allowed us to identify subset of genes with very large sequence similarities and therefore may derive from a common ancestor. In all cases we also used GO slim annotations [15] as a guidance for genes likely functionality. Aimed to get a fair share of representatives from all functional and phylogenetic gene sets, we randomly sampled genes from each set such that the number of samples was proportionally to the set size. We ended up with approximately 500 background genes for case study 1, and 600 genes for case study 2 and 3.

4) Identify homologous genes in nearby species (case study 1: *S. Japonicus* and *S. Octosporus*; case study 2 and 3: *C. Tropicalis*, *C. Dublinensis* and *C. Parapsilosis*) for both the co-regulated and background sets. We used a recent homology map [16] to facilitate this step.

5) Extract upstream DNA sequences (1000 base pairs length) for each species, shorten them in case of overlapping with adjacent ORFs. For genes with negative orientation, we considered the reverse complement of the sequences. Note that motif finding algorithms are sensitive to noise, which increases with the size of upstream sequences examined, moreover the vast majority of the yeast regulator sites from the TRANSFAC database are located within 800 bp from the translation start sites [17].

### Selection of candidate motifs

6) Generate candidate motifs enriched in promoter regions of co-regulated genes and compute their matching scores for each gene. We used a modified version of the software MDSCAN [7] to search for nucleotide patterns which appears in the upstream sequences of the genes of interest for each species. To remove repeated segments that might confuse the motif discovery process, we preprocessed the upstream sequences of the interested network genes using RepeatMasker [18]. The matching score between a candidate motif 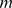 and a given gene sequence 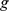 was calculated as in [6]:
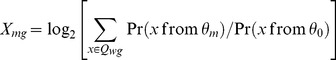
where 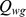 is the set of all 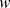-mer in the upstream region of gene 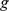. 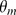 is the probability matrix of motif 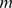 of width 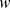, 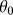 is the transition probability matrix for the background model, computed using a Markov chain of the sixth order (Liu's original algorithm permits only Markov chain of the third order) from the upstream regions of all the species of interest. We examined nucleotide patterns of length 5 to 12 bp and scored up to 30 distinct candidates for each width in all case studies.

### Variable Selection and Inference

7) Identify likely regulatory motifs among the candidate sets obtained in the previous step. We used an extended version of the Bayesian variable selection [9] that handles the case of multiple experiments [19]. The idea is to search for the set of motifs that provide the best fit when regressing nucleotide pattern matching scores (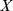) to the set of gene expression levels (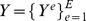). 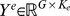 contains the expression data from experiment 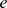 of 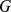 genes of the annotated species over 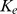 technical replicates. Pattern scores 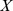 is of size 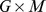 where 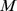 is the number of candidate motifs, evaluated on the nearby species. We assume the following model
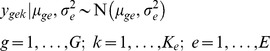
(1)where 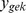 represents the observed gene expression value of the gene 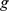 in the 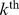 replicate of the 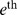 experiment and 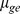 is the underlying transcriptional activity level of gene 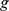 under the experimental condition 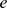. A binary latent vector, 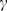 of dimension 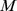, is introduced to indicate the inclusion of variables in the model; 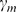 takes on the value of 1 if the 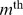 variable (motif) is included and 0 otherwise. Let 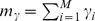 be the total number of motifs included. The true gene expression value 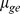 is connected to a specific subset of the 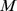 candidate motifs identified by the latent vector 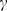 by the following relation
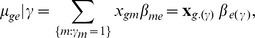
where 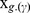 is the row vector of matching scores for all included motifs against gene 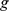. We specified the following priors for the regression coefficients, the experiment variance, and the latent indicator:
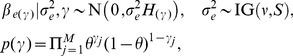
(2)where prior parameters 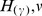 and 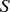 were assessed by a sensitivity analysis and 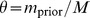 with 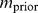 as the number of covariates expected *a priori* to be included in the model. For all case studies we chose 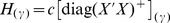, where 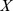 is the score matrix obtained in step (6) and 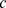 equals to the variation of the regression coefficients of the full model averaged over the experiments. We set weak prior knowledge choosing 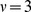, which is the smallest integer such that the expected noise level of an experiment 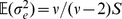 exists. The scaling value S is equal to data variation averaged over all experiments. For case study 1, whose data are from time-course experiments, 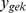 represents the average value of gene expression levels measured in the interval when the ENG1 genes show their common activity peak, approximately 30–90 minutes. The model specified in equation (1) could be rewritten as
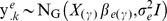
where 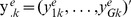, 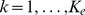 and 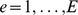. Without loss of generality we further assume that the columns of 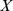 and 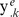 are mean-centered.

Having set the prior distributions, a Bayesian analysis proceeds by updating the prior beliefs with information that comes from the data. The posterior distribution of the latent indicator vector 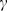 given the data, i.e., 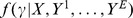, can be obtained:
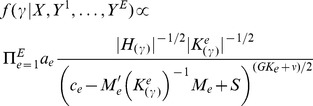
(3)with
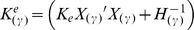


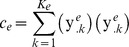


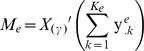


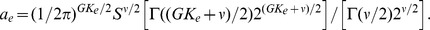



The model (1)–(2) could be generalized in order to handle the presence of missing data which are typically encountered when analyzing real data experiment. For each fixed experiment 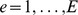 and each fixed technical replicate 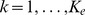, let 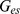 be the number of genes with expression levels measured on the array. In this case the posterior becomes
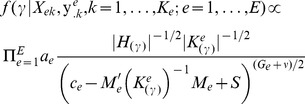
(4)with
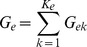


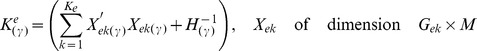


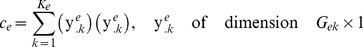


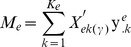


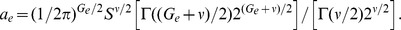



Our interest is to maximize the posterior probability in equations (3) and (4). Since the relative high dimensionality of our vector space (approximately 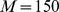 for our case studies) makes comprehensive evaluation of posterior probabilities impossible, we employed a sampling procedure based on stochastic search Markov Chain Monte Carlo (MCMC) technique to identify realizations of 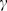 with huge posterior probabilities.

8) Run multiple parallel MCMC chains of significant length for each species. In each run, the algorithm visits a sequence of models that differ successively in one or two variables. At each iteration, a candidate model, represented by 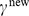, is generated by randomly choosing one of these two transition moves:

- Add or delete one variable from 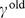.
- Swap the inclusion status of two variables in 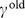.

The proposed 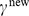 is accepted with a probability that depends on the ratio of the relative posterior probabilities of the new versus the previously visited models:
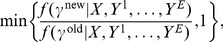
(5)which leads to the retention of the more probable set of patterns. An analogous formula is obtained considering the posterior probability given by formula (4).

The stochastic search results in a list of visited sets (i. e. combination of candidate motifs) and the corresponding relative posterior probabilities, then the selection of few “best motifs” can be done either using the global MAP principle or by selecting the covariates on the basis of their marginal probability to be included. The marginal posterior probability of inclusion for a single motif 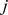, 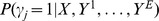, can be computed by averaging out the posterior probabilities of the acquired samples:
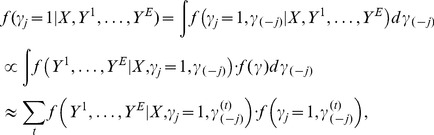
(6)where 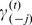 is the vector 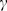 at the 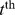 iteration without the 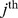 motif.

In all three case studies, we ran 10 parallel chains of 100,000 iterations each. We computed the normalized posterior probabilities for each distinct visited set of motifs and the marginal probabilities for the inclusion of single nucleotide patterns.

### Robustness analysis

To investigate the effect of sparsity setting on variable selection, we ran steps 7)–8) with various values for 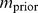, the a-priori expected number of motifs included in the model. In particular we examined 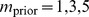 for all three case studies. These values were chosen due to the knowledge that fungi are simple organisms and their regulation mechanisms are based on relatively few motifs.

To study the robustness of the proposed framework with respect to the choices of both single experiment and control gene sets, we repeated steps 7)–8) with different subsets of control genes in combination with the leave-one-out cross validation strategy over all experiments. In our case studies we randomly sampled 8 different subsets of 200 genes out of 500–600 background genes in total.

### Internal testing analysis

The procedure described above is based on the implicit assumption that the expression levels of the co-regulated genes observed in a microarray experiment 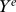 of one species are positively correlated to those of the homologous gene set in the species of interest. In the case experimental data are not available for the latter species, we could validate such assumption using a third species of larger phylogenetic distance than the species under studies. Let 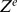 be the expression data of the third species, we could compute the correlation coefficient of the co-regulated gene set in 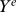 and 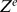. If the coefficient is significantly high (i.e. close to 1), we deduce that the assumption is likely to be satisfied. In this particular context for the first dataset we computed the correlation coefficients between *S. Pombe* and *S. Cerevisiae* in order to justify the comparison of the networks of *S. Pombe*, *S. Octosporus* and *S. Japonicus*. This approach requires a good degree of agreement from several species as criterion of trust of the solution.

### Further generalisation

Our method can be generalized to handle the case when multiple experiments from different species close (phylogeneticaly) to the species under investigation are available. A straightforward solution is to run the described model independently for each species and pool out proposed models by their marginal probabilities. An alternative proposal is to incorporate all information available into a single model as follows.

Let 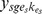 be the observed gene expression value of the gene 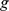 in replicate 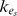 of experiment 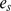 from species 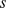, with 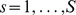; 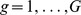, 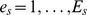, and 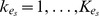. As before, we assume expression values follow a normal distribution,
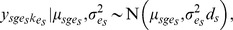
where 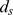 is proportional to the distance between species 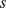 and the species of interest and is estimated from the phylogenetic tree. The distances are normalized such that 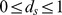 and 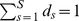. Let 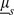 be a matrix of dimension 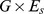, which is defined as
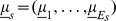
where 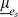 is a column vector of length 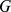 and represents the genes expression values in the 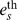 experiment for species 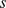. We further assume a normal matrix variate distribution on 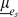 as follows:
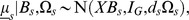
where 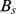 is the coefficient matrix of dimension 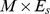, 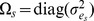 is the covariance matrix of dimension 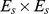 over experiments, and 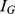 is the identity matrix of dimension 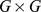 over genes. We assume varying noise levels over experiments, but fixed global noise for biological systems. Conjugate priors are employed for the coefficients 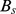 and the covariance matrix 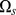:
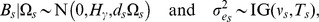
where 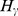 is the covariance matrix for the motifs of dimension 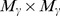, assumed to be known a priori, and 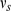 and 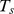 depends on the species 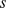. By integrating out 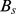 and 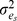 and computing the posterior 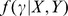, inference can be performed similarly to the previously described procedure.

## Results and Discussion

### Case Study 1: septation transcriptional network in fission yeast clade

One of the key biological processes in the cell is the cytokinesis during which daughter cells separate and form two independent entities. In many unicellular fungi such as the fission yeast *Schizosaccharomyces Pombe*, a contractile actomyosin ring (CAR) generates a cell cleavage and the newly synthesized membrane is inserted at the division site. *S. Pombe* cells then divide by medial fission through the contraction of an actomyosin ring and the deposition of a multilayered division septum that must be cleaved to release the two daughter cells. Seven genes (adg1, adg2, adg3, cfh4, agn1, eng1, and mid2) whose expression is induced by the transcription factor Ace2p have been identified. Their transcription levels vary during the cell cycle, while maximum transcription are observed during septation [20], [21]. The division septum has a threelayer structure, with a central primary septum (mainly composed of linear 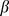-1,3-glucan) surrounded on both sides by two secondary septa (composed of 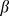-1,6- branched 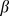-1,3-glucan and 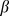-1,6-glucan). The primary septum is synthesized through action of the Cps1/Bgs1 glucan synthase, while Bgs4 is involved in the assembly of secondary septa. Daughter cell separation requires an enzymatic process that controls the degradation of the components of the primary septum and the surrounding cell wall. To date, the two main enzymatic activities identified are exerted by the endo-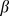-1,3-glucanase Eng1, which is responsible for primary septum hydrolysis, and the endo-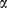-1,3-glucanase Agn1, which is necessary for the erosion of the cylinder of the cell wall surrounding the septum. The pattern of activation of Eng1 involves Sep1p, a protein of the conserved forkhead family. This protein targets a gene which encodes the transcription factor, Ace2p. Two of the Ace2p target genes encode proteins with known roles in cell separation: the 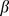-glucanase Eng1p, that degrades the primary division septum between the new ends of daughter cells, and the 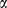-glucanase Agn1p, that hydrolyses the old cell wall surrounding the septum leading to full separation of daughter cells [22]. Cells that constitutively overexpress Ace2 become round and show high transcript levels for both Eng1 and Agn1. The round shape of these cells could reflect a weakening of cell wall material that is not associated with the division septum, caused by an overproduction of glucanases [23], [24]. Both [25] and [22] have found the motifs CC(T/A)CG(T/C)TCC, and (A/T)ACC(T/A)CGC(T/A). Interestingly, the consensus site for Ace2 (CCAGCC) is reminiscent of the core of New 3v (CCACGC), suggesting that an unknown Ace2-like factor could be involved.

We applied the Bayesian variable selection framework (as described in the Materials and Methods section) to detect binding motifs that regulate the network through various cell cycle phases. We obtained expression data from experiment elutriation A described in [25] and experiments elutriation 1, elutriation 2, elutriation 3 and cdc25 block release 1 described in [22]. The experiments explore the transcriptional activity of the fission yeast *S. Pombe* as a function of time in cells synchronized by different approaches: centrifugal elutriation and the use of temperature sensitive cell cycle mutants. All these experiments have no technical replicates. The upstream sequences for *S. Pombe*, *S. Japonicus* and *S. Octosporus* were obtained from the MIT Broad Schizosaccharomyces database [26].

**Figure 1:**
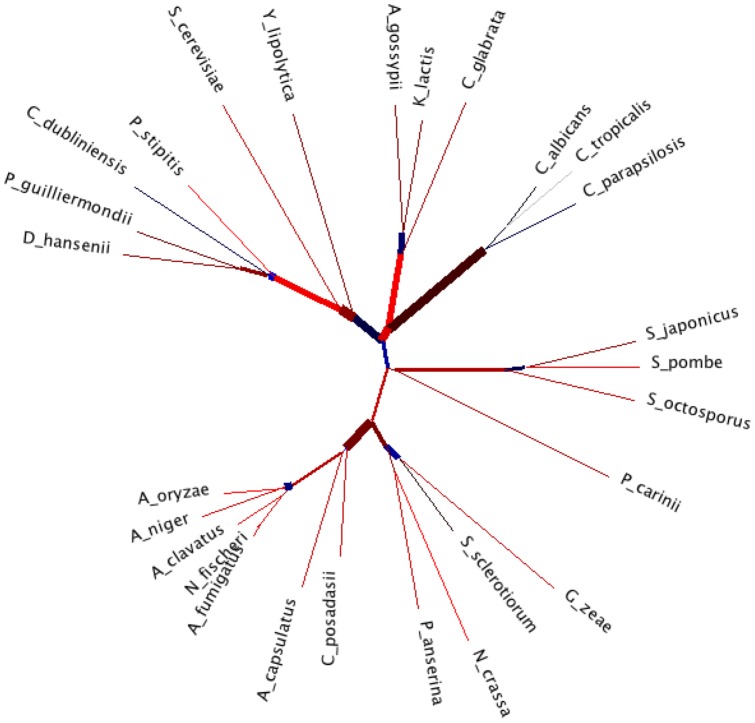
ENG1 ML tree. Maximum Likelihood tree, based on JTT model of evolution, inferred using Eng1 protein sequence from the following species: *S. Japonicus*, *S. Octosporus*, *S. Cerevisiae*, *S. Pombe*, *Kluyveromyces lactis*, *Debaryomyces hansenii*, *Candida Albicans*, *Yarrowia lipolytica*, *Aspergillus oryzae*, *Phaeosphaeria nodorum*, *Neurospora crassa*, *Vanderwaltozyma polyspora Neosartorya fischeri Pichia guilliermondii*,*Coccidioides posadasii*, *Gibberella zeae*, *Ashbya gossypii*, *Sclerotinia sclerotiorum*, *Magnaporthe grisea*, *Ajellomyces capsulatus*, *Aspergillus clavatus*, *Aspergillus niger*, *Pichia stipitis*, *Lodderomyces elongisporus*, *Candida glabrata*,*Candida Tropicalis*,*Candida dubliniensis*,*Candida parapsilosis*; *Brassica napus* and *Sorangium cellulosum* are plant sequences used as outgroups, i.e. to facilitate the rooting of fungi phylogeny; we also include *S. Japonicus* Eng1 and Eng2 proteins and *S. Cerevisiae* Acf1 and Acf2 proteins. From a methodological purpose, we validate this phylogeny with a phylogeny with the same number of species, based on cdc5, a regulator of G2/M transition of mitotic cell cycle with the same visualisation as in [28]; the width corresponds to phylogenetic agreement.

**Figure 2:**
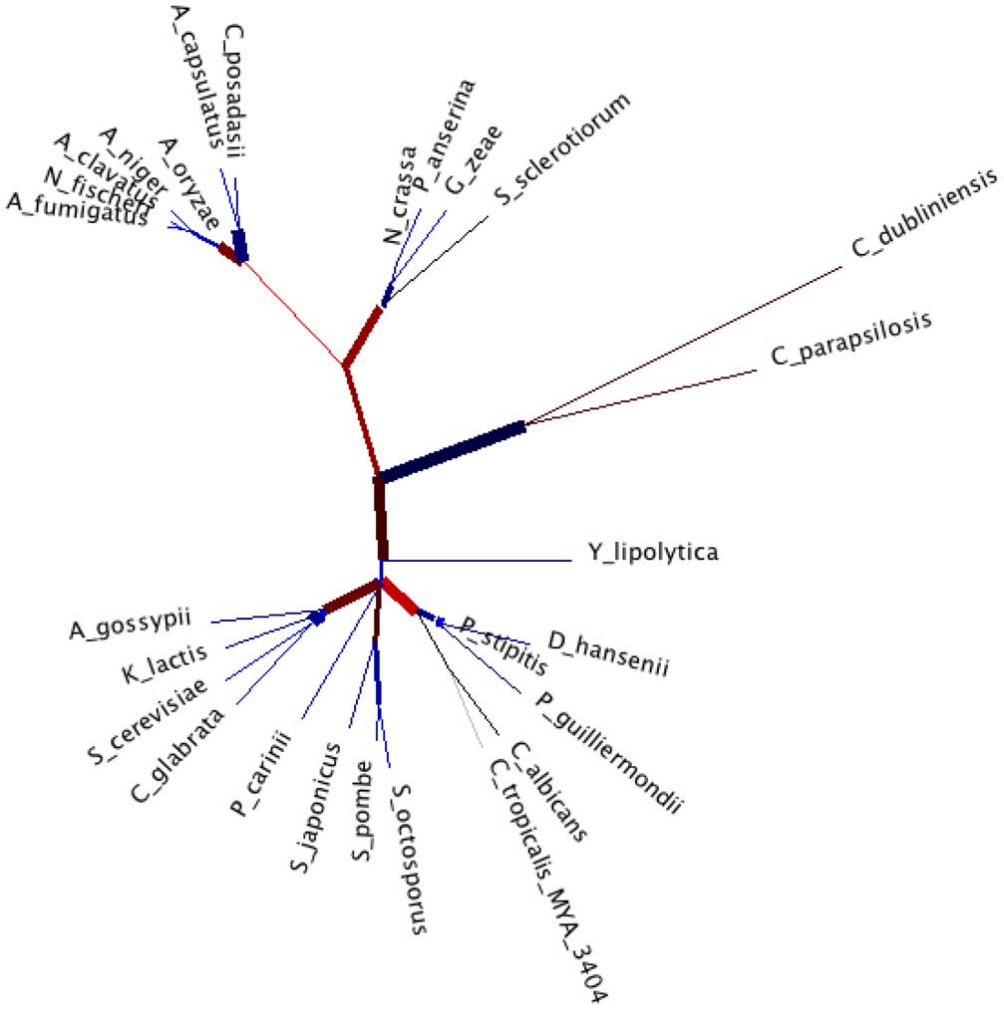
RAM ML tree. Maximum Likelihood tree, based on JTT model of evolution, inferred using RAM protein sequences from the species of figure 1. We validate this phylogeny with a phylogeny with the same number of species, based on cdc5, a regulator of G2/M transition of mitotic cell cycle with the same visualisation as in [28].

Motifs detected with corresponding marginal probabilities larger than 0.5 are shown in Table 1. For the sake of space we present only the results obtained using all the 5 experiments for 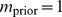. Marginal probabilities were averaged over 8 subsets of control genes. We obtained both confirmation of known results and new findings (motifs) which have high marginal probability values. We note that long patterns were selected more often than short ones. This could be explained by the limited ability to reject associations among nearby DNA bases of the background model. Eukaryotic DNA is highly heterogeneous, patchy and repetitious [27] and currently used genome background models cannot adequately take into account the variations in base association. We also observe that given more replicates or data from more species, the marginal probabilities become much higher (about three-fold) than those obtained using single replicate and one species, see [8]. A good understanding of how genes involved in this network differ in nearby species is provided by phylogenetic inference. Figure 1 shows the maximum likelihood phylogenetic tree obtained using ENG1 protein from a large number of fungi species (see legend), including *S. Japonicus*, *S. Cerevisiae*, *S. Octosporus*, *S. Pombe*, *Candida albicans*, *Candida glabrata*,*Candida tropicalis*,*Candida dubliniensis*,*Candida parapsilosis*. The number of genes of this network ranges from 8 in *S. Cerevisiae*, *S. Pombe*, *S. Japonicus* and *S. Octosporus* to 4 in the candida species. The tree shows ticker lines for worst match with respect the tree of Figure 2 (RAM network, case study 3), with overall topological score of 61.5, see [28] for details. To explore the capability of our approach in comparison to other regression-based motif discovery methods, we applied MatrixREDUCE [12] to the same sets of sequence and expression data. MatrixREDUCE also exploits the correlation between gene expression levels and the occurrence frequency of short DNA segments in upstream sequences to discover binding motifs, but differs from our framework in two following points. Firstly, it employs a deterministic forward variable selection scheme. Motifs are added to the regression model looking at their matching coefficients in descending order. Although this approach is attractive by its computational simplicity, it is more likely to get trapped in local maxima than the stochastic sampling process employed by our procedure. Secondly, our proposed approach provides a principled way to account for multiple experiments/replicates simultaneously while MatrixREDUCE is applicable to one experiment condition at a time. From the results in table 1, MatrixREDUCE could detect only parts of the consensus motif sequences in *S. Pombe* and *S. Japonicus*. We show in the supplementary material the motif marginal probabilities for 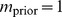 of *S. Pombe* (Figure S1a in the supplementary material), *S. Japonicus* (Figure S1b in the supplementary material), *S. Octosporus* (Figure S1c in the supplementary material).

**Table 1:** Motifs detected for septation transcriptional network in fission yeast clade (case study 1).

Motifs detected in *S. Pombe*			
by Bayesian variable selection	marginal probability	by MatrixREDUCE	t-value
GGT **GGCTGG**CA	0.995872	**CCAG**	−8.495
AATGTAA	0.992299833	AT	7.854
GTGGTTGG	0.990841403	TCTG	−7.146
TTGCTTTAT	0.966439608	GAGAA	−6.868
GAAAATCGAA	0.964823733	CCTC	−6.416
ATCGATGGTAA	0.964302733	TCCTC	−6.119
CAAGAAAGTAC	0.952851275	CG	−6.017
TCAATAT **CCAGC**	0.930580914	AT	5.857
GATTTTACCA	0.930109523	CTCT	−5.723
TTAT **CCAGCC**	0.913970665	TTC	−5.602
GTAAAAAA	0.911840485		
AAATTTAAGAG	0.899786547		
TTATATAA	0.892315745		
CAAATATAAA	0.8920356		
CATGGCGGG	0.868056013		
TCTATATTCGG	0.773856698		
TTACTTTCTT	0.728913078		

Motifs detected in *S. Japonicus*			
by Bayesian variable selection	marginal probability	by MatrixREDUCE	t-value
ACTCGCGTCAC	0.962531425	A **CAGC**G	−15.946
AAGGA **GGCT**	0.88789641	GCAT	8.422
ACGGTGTGAA	0.860245247	TCGGT	−7.192
**GGCTGG**	0.8282509	TCGGT	−5.905
A **GGCTGG**T	0.789977544	TTTTCC	−5.707
CAGATTTCGTGC	0.777363805	TCGGT	−5.457
A **CCAGCC**	0.742744755	TTTTTT	−5.412
GTGTCAC	0.72705333	AGGA	5.361
ATGCATA	0.70449432	TTTTT	−5.351
		CTAA	5.292

Motifs detected in *S. Octosporus*			
by Bayesian variable selection	marginal probability	by MatrixREDUCE	t-value
GAT **GGCTGG**TA	1	CG	−8.395
GTATCGGTTG	0.998044545	TCGAA	−7.167
GTTGCAAGT	0.997534734	CTTGA	7.12
TTGTTTGTTTA	0.99644855	AGACA	−7.12
ACTTTCATCCA	0.99155467	AGATTT	−6.574
ACAATGGAT	0.98432507	AGGC	−6.574
CCTTCCACCGA	0.980347067	AAGAT	−6.17
TCAAT **CCAG**T	0.975195473	GATCA	−5.87
TTCGTTTCCGT	0.955906395	ACTGAA	−5.287
CATTCAGGGG	0.955746803	AAGATTT	−5.287
ACTTTACTC	0.92077283		
AGAGAGAAA	0.91857454		
GGTACGAAGAA	0.911070636		
AAGAGCAGAGC	0.88196748		
CGTCGTGGTG	0.870479665		
GTTCGATGGC	0.83167287		
GATTTTACTCG	0.7601617		
GTAGAAACA	0.757162374		

### Case study 2: cytokinesis transcriptional network in *Candida* clade

In the second case study we consider the cytokinesis process of *Candida albicans* in the context of three other closely-related species *Candida dublininensis*, *Candida tropicalis*, and *Candida parapsilosis*. While the *Candida* clade is in close approximation to the fission yeast *S. pombe* in the phylogenetic tree, it does not go through the genome duplication event.

The cytokinesis and mother-daughter cell separation processes of *C. albicans* have been associated with 94 genes, whose expression levels periodically peak during the G2/M phase over four biological replicates [29]. Using this gene set as the starting point, we applied hierarchical clustering with Bayesian similarity measurement [30] on their expression profiles to separate regulatory networks with different transcription factors. Eight tightly co-regulated genes were chosen for our further analysis: Cdc5, Chs1, Hof1, Kip2, Chs8, Fgr29, and two less known genes orf19.1334 and orf19.6119. Among the selected genes, the first five are known to preserve their functions as compared to *S. cerevisiae*, and the latter three are specific to *C. albicans* only, without any orthologs in the other two model budding and fission yeast.

We applied Bayesian variable selection and MatrixREDUCE to detect binding motifs in the four *Candida* species using time-series microarray expression data of *C. albicans* and upstream sequences from all four species. The expression data were collected from four independent biological replicates [29]. The upstream sequences for *C. Albicans*, *C. Tropicalis*, and *C. Parapsilosis* were obtained from the MIT Broad Candida database [26] , and *C. Dubliniensis* from Sanger Institute Sequencing Project [31]. We show a summary of the results obtained using all four experiments for sparsity setting 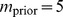 in Table 2. While motifs with longer width are treated more favorably, the algorithm is able to pick up a number of patterns. Two emergent patterns that occur repeatedly in all four species are TCATTC and TCAATT (which were printed bold in the tables for easier comparison). These are suggestively the variants of the consensus motif TCA(A/T)T(C/T). The results from MatrixREDUCE are also presented in the same table. In agreement with the discussion of method comparison in the first case study, Bayesian variable selection definitely adds significant value from its comprehensive combinatorial effect search. While MatrixREDUCE could identify some parts of the proposed motif in *C*. Albicans, it fails to do so for the other related species. Note that *C. Parapsilosis* has been observed in clinical literature [32], [33] to have distinct features in comparison to *C. albicans*, *C. Dubliniensis*, and *C. Tropicalis*. While the other three species are strictly human pathogen, *C. Parapsilosis* is also found in a wide range of environments including animals, soils, and the sea. Such flexibility might suggest corresponding shift in its cell cycle regulatory mechanism. We show in the supplementary material the motif marginal probabilities for the following species: *C. Albicans* (Figure S2a: 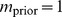; Figure S2b: 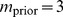; Figure S2c: 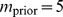), *C. Dubliniensis* (Figure S3a 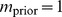; Figure S3b 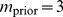; Figure S3c 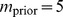), *C. Tropicalis* (Figure S4a 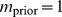; Figure S4b 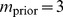; Figure S4c 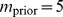), *C. Parapsilosis* (Figure S5a 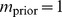; Figure S5b 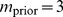; Figure S5c 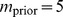).

**Table 2:** Motifs detected for cytokinesis transcriptional network in Candida clade (case study 2).

Motifs detected in *C. Albicans*			
by Bayesian variable selection	marginal probability	by MatrixREDUCE	t-value
T **TCATTC**ATTC	0.978005	AATGAA	10.56
**TCAATT**	0.900631	AT **AATT**	−8.362
**TTGA**T	0.666508	AAATGAA	8.143
TGAAATCA	0.663159	TGAAAT	7.691
ATGAAATA	0.650006	**CAAT**	7.129
GAAACTGA **AATT**	0.637142	AAGTT	6.78
**AATT**AATT	0.626425	AATGAAT	6.09
		GTTGTTG	6.09
		ATGAA	6.086
		**AATTA**AT	6.086

Motifs detected in *S. Dubliniensis*			
by Bayesian variable selection	marginal probability	by MatrixREDUCE	t-value
GTT **AATT**CCA	0.999259	ATTT	8.486
AGTTCA	0.962802	CAACAA	8.067
TTTCCTGATTTG	0.925764	CATCA	−7.891
CCTA **AATT**AAG	0.870212	GTCT	7.473
AAT **TCAATT**	0.82158	ACAACAAC	7.471
TCCTGA	0.804322	CAAAATA	7.192
TATGCAA	0.68007	AGG	−7.101
**TCATTC**CACTT	0.58234	CAACAACA	6.904
**TCAAT**	0.56146	CAACAAC	6.058
TCCTGATTTG	0.560856	ACAACAA	5.94
TTCGTC	0.548985		

Motifs detected in *S. Tropicalis*			
by Bayesian variable selection	marginal probability	by MatrixREDUCE	t-value
TAATG **CATT**	0.74355	CCATG	12.846
**AATT**T	0.699152	TATTTAT	11.433
TAATGAAA	0.684862	TTATTTAT	10.903
TGAAACT**TTGA**A	0.662662	TTATTTA	10.267
ATTTGGTCA	0.562592	TTTATTTA	9.598
		TATTTATT	8.848
		ATTTAT	8.701
		ATTTATTT	8.645
		TAACA	8.432
		GTTGGT	−8.432

Motifs detected in *S. Parapsilosis*			
by Bayesian variable selection	marginal probability	by MatrixREDUCE	t-value
AT **TCAAT**	0.671743	AGAGA	10.182
GC **CAATT**C	0.548763	CCAT	−9.971
AGATAAGCA	0.547218	AGAG	7.773
TTCCA **AATT**	0.54143	GAGAT	7.626
		GAGA	7.356
		AGAGAG	7.26
		AAAAC	−6.469
		CAAACA	−6.384
		AAAC	−5.884
		CTA	−5.504

### Case study 3: RAM transcriptional network in the *Ascomycota*

RAM (regulation of Ace2p transcription factor and polarized morphogenesis) is a conserved signaling network that regulates polarized morphogenesis in yeast, worms, flies, and humans [34]. In unicellular fungi, the RAM network comprises the proteins Cbk1, Mob2, Kic1, Hym1, Sog2, and Tao3. *S. Cerevisiae* strains harboring mutations in any of these genes display cell separation defects and a loss of polarity. The ability of *C. Albicans* to undergo morphogenesis from yeast to hyphal form is associated to the condition of causing the disease; the RAM genes CaCBK1, CaMOB2, CaKIC1, CaPAG1, CaHYM1, and CaSOG2 are good candidates for drugs controlling the growth of the organism and therefore the spreading of the infection [35]. Figure 2 shows the maximum likelihood phylogenetic tree obtained using CaCBK1 gene sequences from the same species of Figure 1.

Microarray data of *C. Albicans* were obtained from a recent investigation of RAM network's role in cell polarity and hyphal morphogenesis processes [35]. Four single-replicate experiments were conducted on wild-type (SC5314) and CaMOB2 mutant strains grown in either normal yeast or hypha-inducing serum medium. The identified RAM-dependent hypha-specific genes suggested the association of RAM network with Tup1p/Nrg1p-regulated morphological processes.

A summary of the results for all four species is shown in Table 3. The left two columns list out motifs detected by Bayesian variable selection with their marginal probabilities averaged over 8 control subsets. The right two columns list out motifs detected by MatrixREDUCE with corresponding T-values. To account for the possible depreciation of motif effects in various species, we lowered the cutting threshold of marginal effect to 0.3 for *C. Albicans* and *C. Dubliniensis*, and 0.2 for *C. Tropicalis* and *C. Parapsilosis*. Two emergent patterns that occur repeatedly in all four species are AAAGA and AAATA, which constitute the consensus motif AAA(G/T)A. Again the comparison with MatrixREDUCE results suggest the capability of our approach to detect more comprehensive patterns. We present the motif marginal probabilities of *C. Tropicalis* for 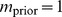 (Figure S6a of the supplementary material); for 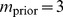 (Figure S6b of the supplementary material) and for 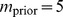 (Figure S6c of the supplementary material).

**Table 3:** Motifs detected for RAM transcriptional network in the Ascomycota (case study 3).

Motifs detected in *C. Albicans*			
by Bayesian variable selection	marginal probability	by MatrixREDUCE	t-value
AATGAG **AAATA**A	1	CCAA	10.494
GAGTTGA	0.7266	CCATA	−10.281
GAGA **AAAGA**AAA	0.7263	GATTAC	8.597
A **AAAT**	0.6174	ATCAC	8.591
ACTTT **TCTT**A	0.4965	CTAAA	8.591
**TATTT**	0.4731	**TATT**GA	−8.383
TGGATTTTG	0.4458	TCATAT	−7.47
AGAC **AAGA**	0.4028	ACTCT	7.47
AAAATGAA	0.3884	AGGC	7.104
CTTT **TCTT**	0.3768	A **ATTT**	−6.759
TTGAC **CTTT**	0.3666		
T **TATT**	0.3254		
**TATT**GGA	0.319		

Motifs detected in *S. Dubliniensis*			
by Bayesian variable selection	marginal probability	by MatrixREDUCE	t-value
**AAAT**GAAAGG	0.982	CTCT	19.105
**AAAT**TCAATTTC	0.7966	ATGTTT	−8.855
GAAAA	0.7782	AGGTG	8.597
AAAAC	0.7464	GGGT	8.431
ATTTC **TATTT**	0.7382	G **TATT**	8.297
TCAGTTTTAA	0.6154	ATGTT	−8.022
TTA **CTTT**TCT	0.6129	TAGG	7.959
TGGTAG **TATT**G	0.3549	TCTCTC	7.945
CTTTT **TCTT**	0.3287	GGTC	7.104
TATATATATATA	0.3155	CTCTC	6.883

Motifs detected in *S. Tropicalis*			
by Bayesian variable selection	marginal probability	by MatrixREDUCE	t-value
AATTAA **TATT**CG	0.9516	C **AATA**	−15.527
GTCTGT	0.8571	TCCAA	10.351
TCTGTAGATAG	0.553	A **AAAT**G	9.408
TACACGAACAAT	0.4903	CAATTA	−8.855
GTAGAGG **AAGA**	0.4588	TC **AATA**	−8.855
CTGTAG	0.3561	TGCAA	8.383
TACACGC	0.2928	TTGC	7.979
ATTGT	0.2789	GTATA	7.959
**AAAGA**GACAC	0.2734	CCAAG	−7.47
ACAAC	0.2242	GGATT	7.47
GACAA	0.2179		
AGTGT	0.2151		

Motifs detected in *S. Parapsilosis*			
by Bayesian variable selection	marginal probability	by MatrixREDUCE	t-value
TGTGGTGGT	0.8894	ATGTG	−12.033
ACATACACCCC	0.5923	GAA	11.016
CAC **AAGA**GCAA	0.3663	TCGC	−10.351
AGTTGATCCTA	0.3434	ACAAAC	10.351
AGTTG	0.3056	GATGT	−10.281
GAGAACCGTT	0.297	TGAAC	8.597
TACAACCC	0.2714	**AAAT**GA	8.597
ACTTG	0.2643	CAAGT	8.383
GGTATGTGTGTA	0.2484	G **AAGA**A	8.112
A **CTTT**AA	0.205	ATGCA	−7.47

### Further points of discussion and Conclusions

Here we present a general algorithm to predict sequence motifs in a newly sequenced species combining linear regression, Bayesian variable selection and experimental data of a well characterized model organism. First we apply our method within the same species (with several replicas), then we extend it to phylogenetically close model species. The proposed method for regulatory motif discovery do not rely on previous knowledge of co-regulated sets of genes, and in that way differ from the main stream literature on computational motif discovery. The validation of the discovered motifs relies on previously published regulatory motifs in yeasts and on an internal testing procedure. Finally, we apply it to make prediction on three important eukaryotic gene networks and compare the results with a currently used method. We believe that the tables of regulatory sequences we present could be useful to genome researchers because the motifs represent putative regulatory sites and commonalities among the studied species.

Most of the species we have considered are recently sequenced with little annotations available. We have searched the proposed sets of motifs in a number of available low eukaryotes genomic resources such as NCBI , http://www.pombase.org/, http://www.broadinstitute.org/annotation/genome/ and others. We found that motifs with the highest marginal probability have been reported in low eukaryotes and plants. For example GTTAATTCCA (motif detected in *S. Dubliniensis*) has been reported being a target sequence in the phenobank in *C. Elegans* database [36]. The motif TCAATCCAGT (found in *S. Octosporus*) occurs in the promoter region of the locus AT5TE39210 of *A. Thaliana*; ACAATGGAT (found in *S. Octosporus*) is conserved in the promoter of several plants such as barley and wheat (CA679037, homologous to DATFAP); GTATCGGTTG (found in *S. Octosporus*) is a motif reported (http://yeastract.com/) in the promoter of YBR242w, which is a protein of unknown function of *S. Cerevisiae* that localizes to the cytoplasm and nucleus. The motif ATCGATGGTAA (found in *S. Pombe*) has been reported to regulate the expression of the GLN1 in *S. Cerevisiae* [37]; ACTTTCATCCA (found in *C. Albicans*) is reported in regulatory elements in promoters of defense genes (GRX480) in *A. Thaliana* [38].

We also find that some of the motifs ((for example TTTCCTGATTTG and AATGAGAAATAA) have been described in databases automatically built by a number of computational tools such as http://www.cisred.org/ [39], ABS, http://genome.crg.es/datasets/abs2005/ [40] and Phylonet (http://stormo.wustl.edu/molee//Motif/). Some motifs produced hits in high eukaryotes or bacteria sequences (not reported because the species are too distant from the fungi); short motifs produced many hits.

We believe that the conservation of the size of the network across the species and its functional role are very important. Indeed, the first two examples, which are both cell-cycle related with relatively conserved gene network size across the species, give a much better result than the third case (RAM network). Given the high cost of performing a large number of experimental replicates, we make the hypothesis that experimental evidences, from species similar to that under analysis, may provide additional statistical support. We can assume that the closer the species to the one under investigation, the better is. The most interesting result presented in the paper is to show that the marginal probabilities become much higher (about 3 fold) than those obtained using single replicates and one species [8]. It is interesting to consider our work in the light of a recent discussion about experimental design in the context of phylogenetic inference [41]. A first simple question is whether the number of genes involved in the transcriptional network is the same for the different species considered or whether it varies when the phylogenetic distance increases. This is an important point raised in [42], where the authors have demonstrated an inverse correlation between the rate of evolution of transcription factors and the number of genes they regulate. For small gene networks, distant species may not provide adequate support and, in general, the distance may depend on the size of the genetic network. Noteworthy, while much effort has been focused on sequencing the genomes of widely divergent species, recently there has also been interest in sequencing the genomes of closely related species, with the target of comparing and contrasting them for subtle differences [41]. There exist different papers to quantify the utility of multiple genomes for the detection of conserved DNA regions ([41], [43], [44], [45]). Margulies et al. [46] described an economically efficient approach to show that low redundancy sequencing of additional genomes is a useful first step in locating conserved regions in the species of interest. Current efforts in sequencing may allow to sequence ‘on demand’ in order to shed light on an important regulatory network under study. We believe that our statistical approach can result of some practical utility for outputting putative regulatory sites and annotating new genomes. We have also found that it highlights interesting statistical problems raised by high throughput data integration, such as sequence and gene expression. We believe that this methodology could be extended to integrate other high-throughput omic data.

## Supporting Information

Figure S1
**Motif marginal probabilities, case study 1.** Posterior marginal probabilities of (a) *S. Pombe* , (b) *S. Japonicus*, (c) *S. Octosporus* candidate motifs for 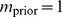.(TIFF)

Figure S2
***C. Albicans*** **motif marginal probabilities, case study 2.** Posterior marginal probabilities of *Candida Albicans* candidate motifs for (a) 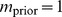; (b) 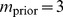; (c) 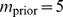.(TIFF)

Figure S3
***C. Dubliniensis*** **motif marginal probabilities, case study 2.** Posterior marginal probabilities of *Candida Dubliniensis* candidate motifs for (a) 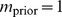; (b) 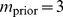; (c) 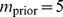.(TIFF)

Figure S4
***C. Tropicalis*** **motif marginal probabilities, case study 2.** Posterior marginal probabilities of *Candida Tropicalis* candidate motifs for (a) 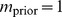; (b) 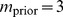; (c) 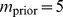.(TIFF)

Figure S5
***C. Parapsilosis*** **motif marginal probabilities, case study 2.** Posterior marginal probabilities of *Candida Parapsilosis* candidate motifs for (a) 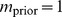; (b) 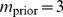; (c) 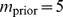.(TIFF)

Figure S6
***C. Tropicalis*** **motif marginal probabilities, case study 3.** Posterior marginal probabilities of *Candida Tropicalis* candidate motifs for (a) 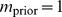; (b) 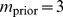; (c) 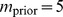.(TIFF)
