# Supplementary figures and images for: Statistical Approaches to Use a Model Organism for Regulatory Sequences Annotation of Newly Sequenced Species
Source: PLoS One. 2012 Sep 11;7(9):e42489. doi: 10.1371/journal.pone.0042489 (PMC3439465; doi:10.1371/journal.pone.0042489)

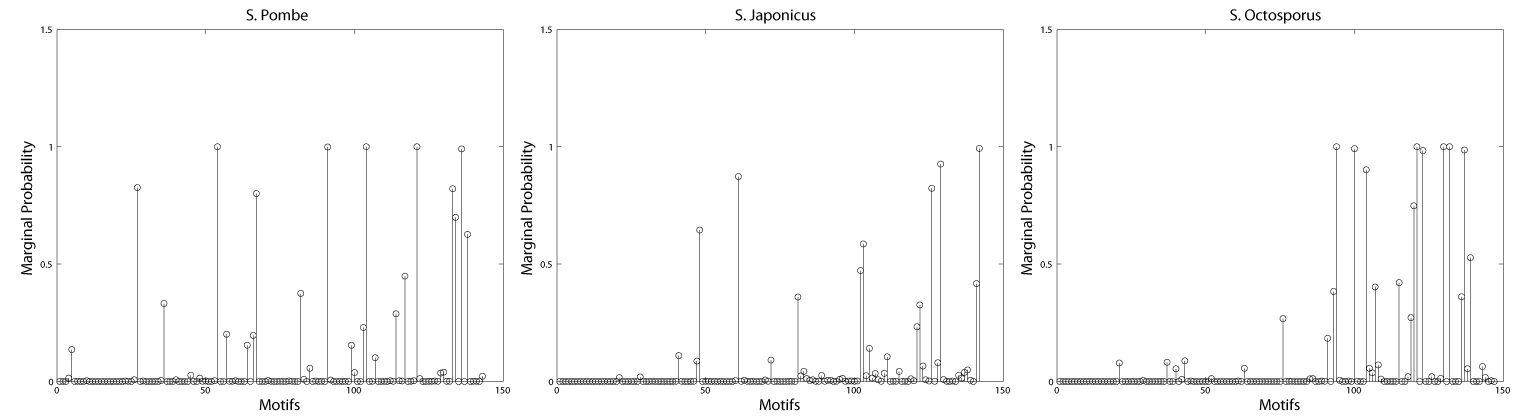

Supplement: Figure S1 — Motif marginal probabilities, case study 1. Posterior marginal probabilities of (a) S. Pombe , (b) S. Japonicus, (c) S. Octosporus candidate motifs for . (TIFF) [file pone.0042489.s001.tiff]

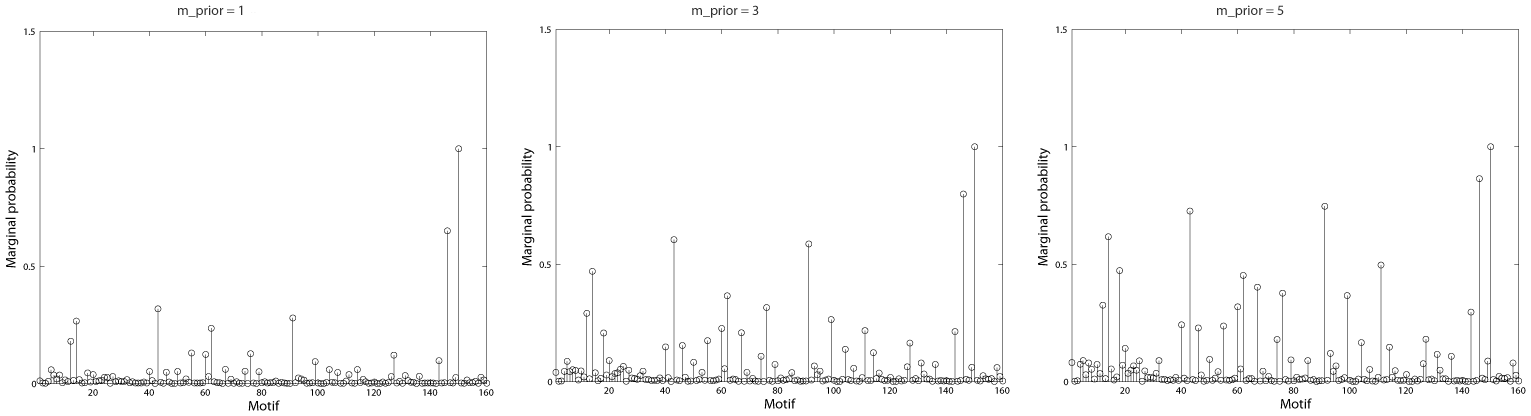

Supplement: Figure S2 — C. Albicans motif marginal probabilities, case study 2. Posterior marginal probabilities of Candida Albicans candidate motifs for (a) ; (b) ; (c) . (TIFF) [file pone.0042489.s002.tiff]

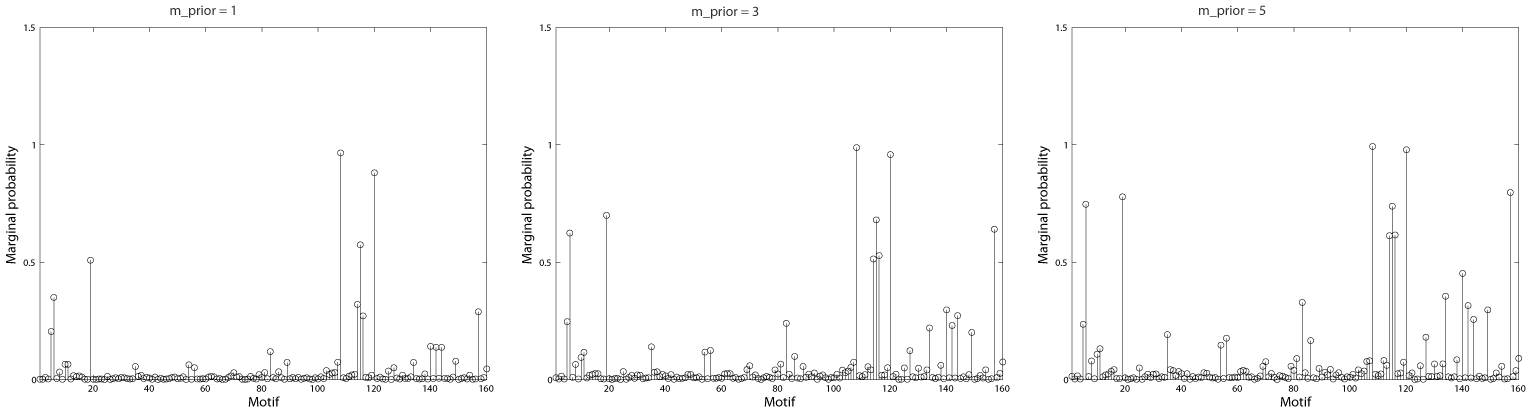

Supplement: Figure S3 — C. Dubliniensis motif marginal probabilities, case study 2. Posterior marginal probabilities of Candida Dubliniensis candidate motifs for (a) ; (b) ; (c) . (TIFF) [file pone.0042489.s003.tiff]

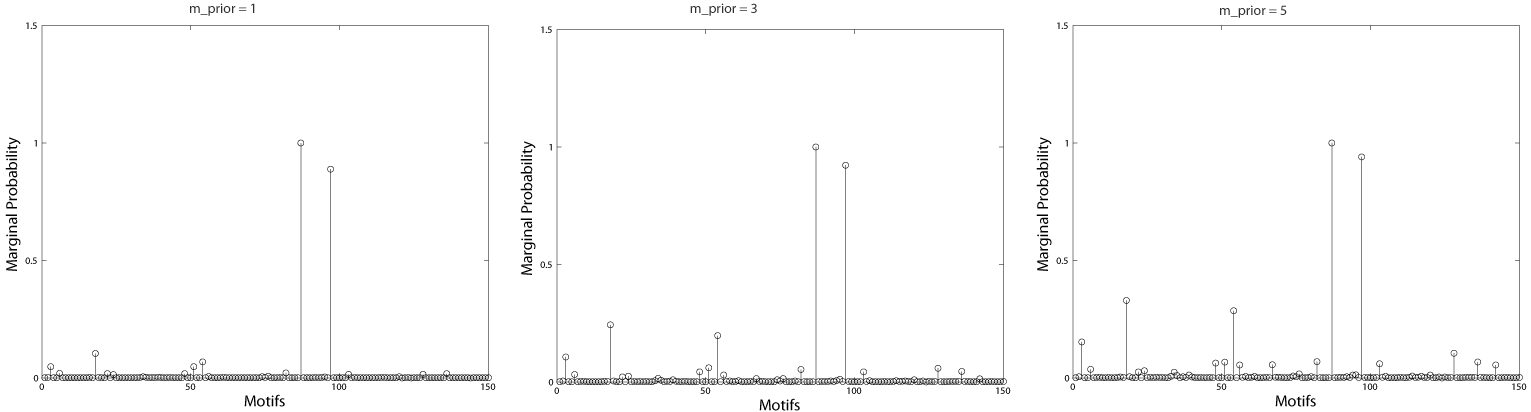

Supplement: Figure S4 — C. Tropicalis motif marginal probabilities, case study 2. Posterior marginal probabilities of Candida Tropicalis candidate motifs for (a) ; (b) ; (c) . (TIFF) [file pone.0042489.s004.tiff]

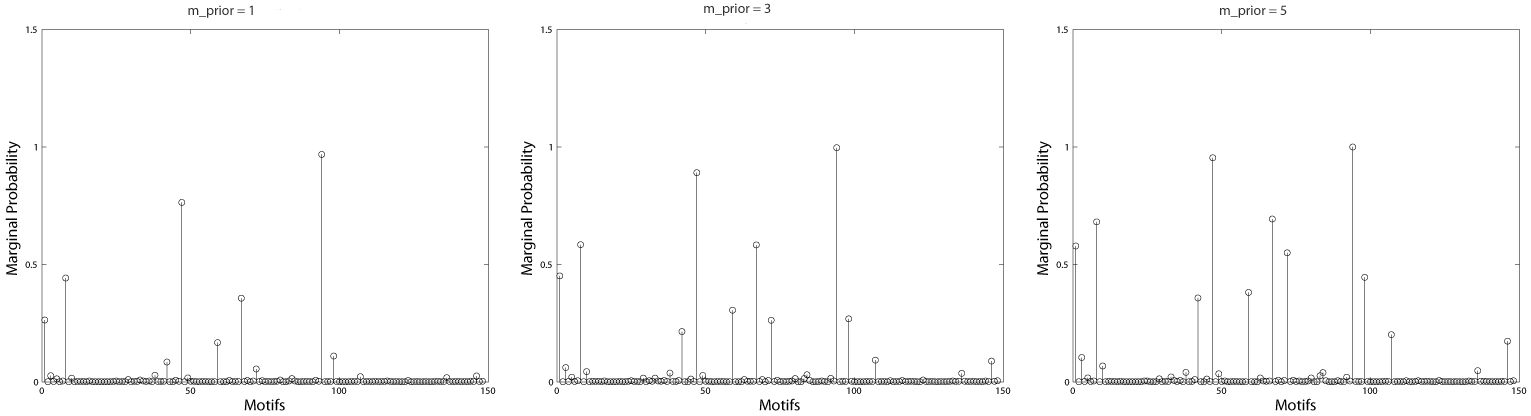

Supplement: Figure S5 — C. Parapsilosis motif marginal probabilities, case study 2. Posterior marginal probabilities of Candida Parapsilosis candidate motifs for (a) ; (b) ; (c) . (TIFF) [file pone.0042489.s005.tiff]

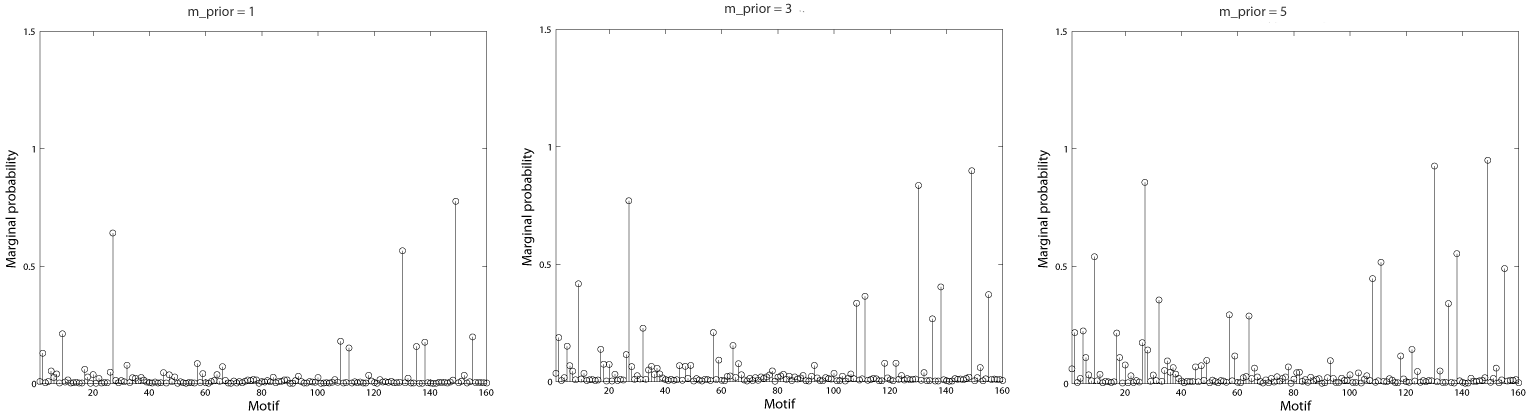

Supplement: Figure S6 — C. Tropicalis motif marginal probabilities, case study 3. Posterior marginal probabilities of Candida Tropicalis candidate motifs for (a) ; (b) ; (c) . (TIFF) [file pone.0042489.s006.tiff]
